# rrvgo: a Bioconductor package for interpreting lists of Gene Ontology terms

**DOI:** 10.17912/micropub.biology.000811

**Published:** 2023-04-18

**Authors:** Sergi Sayols

**Affiliations:** 1 Bioinformatics Core Facility, Institute of Molecular Biology, Mainz, 55128, Germany.

## Abstract

Gene Ontology (GO) annotation is often used to guide the biological interpretation of high-throughput omics experiments, e.g. by analysing lists of differentially regulated genes for enriched GO terms. Due to the hierarchical nature of GOs, the resulting lists of enriched terms are usually redundant and difficult to summarise and interpret. To facilitate the interpretation of large lists of GO terms, I developed rrvgo, a Bioconductor package that aims at simplifying the redundancy of GO lists by grouping similar terms based on their semantic similarity. rrvgo also provides different visualization options to guide the interpretation of the summarized GO terms. Considering that several software tools have been developed for this purpose, rrvgo is unique at combining powerful visualizations in a programmatic interface coupled with up-to-date GO gene annotation provided by the Bioconductor project.

**Figure 1. Different visualizations of the reduced terms provided by rrvgo:**
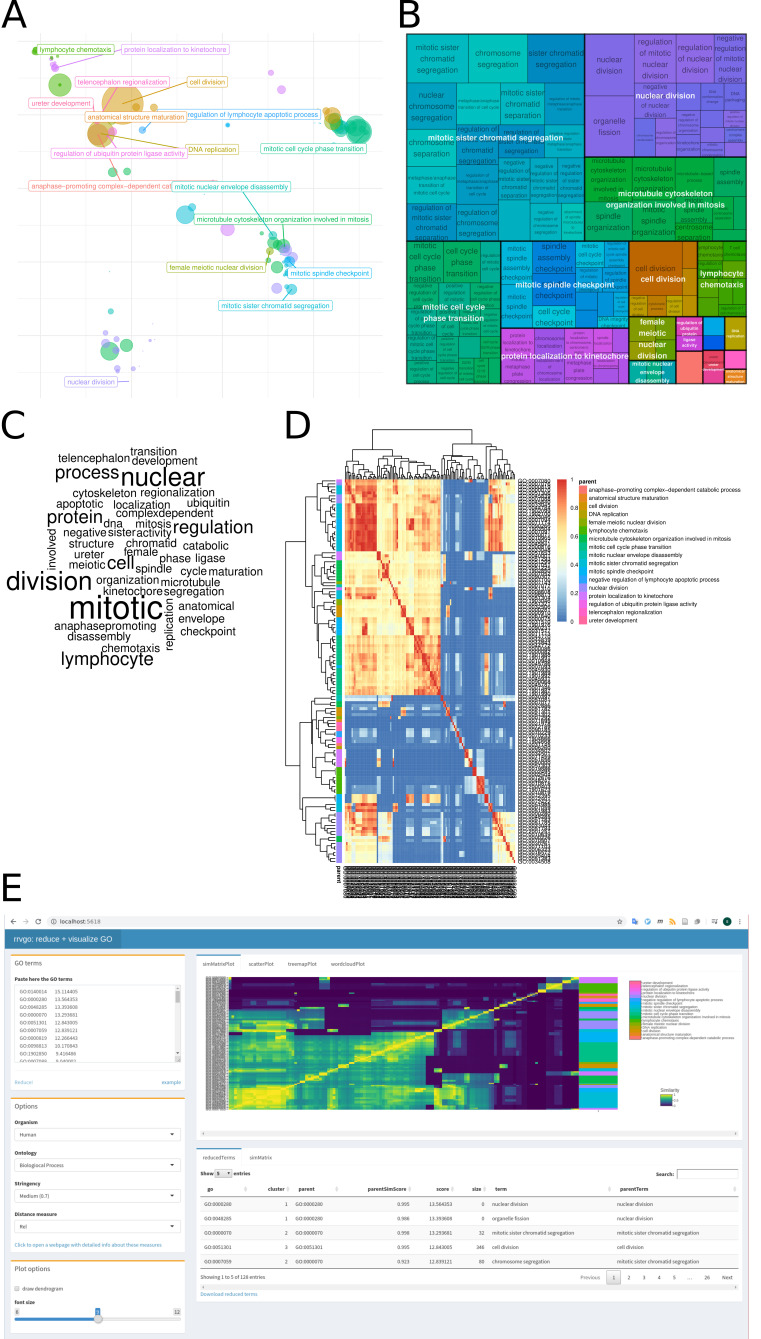
(A) scatter plot represented by the first 2 components of a PCoA of the dissimilarity matrix. (B) space-filling visualization (treemap) of terms grouped by the representative term. (C) word cloud emphasizing frequent words in GO terms. (D) heatmap representation of the similarity matrix. (E) Companion Shiny App for interactive visualization of similarity between GO terms.

## Description

***Introduction***

Structured vocabularies such as GO (The Gene Ontology Consortium. 2019) are important tools for the biological interpretation of high-throughput omics experiments. Due to the hierarchical nature of GO annotation, lists of enriched GO terms are usually large and redundant. One approach to simplify GO analysis is to use GO Slims (Carbon et al. 2009) representing a subset of the full GO. However, using such limited GO versions may hide interesting findings represented by more specific terms which were excluded. Hence, methods such as semantic similarity may better account for the complex structure of the GO graph and be more effective (Pesquita et al. 2009) .

Several online tools to compute semantic similarity between GO terms exist, such as REVIGO (Supek et al. 2011) . The accessibility of such tools comes at a price: they usually offer a limited programmatic interface difficult to integrate into pipelines, and provide pre-packaged GO annotations which cannot be overridden. Offline tools also exist, such as clusterProfiler (Yu et al. 2012) or ViSEAGO (Brionne et al. 2019) including useful but limited exploration capabilities.

Conveniently, the Bioconductor project (Huber et al. 2015) implements several semantic similarity methods and provides up-to-date GO annotations for a number of model organisms, along with the possibility of preparing custom annotations. I developed rrvgo to integrate in a single package access to the semantic similarity methods and annotations implemented in the Bioconductor project, coupled with highly effective visualizations, providing a one-stop-shop for the interpretation of large lists of GO terms in R.

***Implementation***

rrvgo requires a list of GO terms, usually identified in an overrespresentation analysis, from any of the three orthogonal taxonomies: Biological Process (BP), Molecular Function (MF) or Cellular Compartment (CC). Each term in the list may optionally include a score (eg. a minus log-transformed p-value). In this case, rrvgo will prefer terms with higher scores to identify the most representative term of a group; otherwise higher-level terms (ie. those comprising more genes) are preferred by default.

rrvgo uses the GOSemSim package (Yu et al. 2010) under the hood, which implements methods to compute semantic similarity between pairs of GO terms, and the OrgDb packages of the organisms of interest provided within Bioconductor.

***Similarity measures***

The application of semantic similarity methods, originally used in Natural Language Processing, to ontological annotation has already been investigated (Lord et al. 2003) . Some of these measures are based on the calculation of the term's Information Content (Resnik 1999; Lin 1998; Jiang and Conrath 1997; Schlicker et al. 2006) or graph-based (Wang et al. 2007) and are implemented in the GOSemSim package.

rrvgo uses the similarity between pairs of terms to compute the matrix of dissimilarities. The terms are then clustered using complete linkage, and the cluster is cut at the desired threshold, picking the term with the highest score as the representative of each group.

***Organisms supported and creating a custom OrgDb***

As of Bioconductor 3.16, there are OrgDb packages available for the most common organisms used in the lab. Consult the OrgDb BiocView for a full list of current OrgDb packages. It is expected that the list fluctuates between versions, but most common species may be very well supported while the project remains healthy.

For organisms not having an OrgDb package in Bioconductor, it is still possible to create custom OrgDb packages using the AnnotationForge package (Carlson and Pagès 2019).

***Visualizations***

rrvgo provides visualizations of the reduced terms as: (i) scatter plot represented by the first 2 components of a PCoA of the dissimilarity matrix; (ii) space-filling visualization (treemap) of terms grouped by the representative term; (iii) word cloud emphasizing frequent words in GO terms; and (iv) heatmap representation of the similarity matrix. Figure 1A -D.

Alternatively, the results can be interactively explored using the companion shiny app ( Figure 1E ).

***Conclusion***

rrvgo is a Bioconductor package that aims at providing a one-stop-shop for the biological interpretation of large lists of GO terms. It integrates access to semantic similarity methods and visualization in coherent and intuitive manner. This software is heavily influenced by REVIGO, mimicking a good part of its core functionality and some of the visualizations. The strength of rrvgo is its programmatic interface coupled with up-to-date GO gene annotation provided by the Bioconductor project.

## Reagents

rrvgo is available as a Bioconductor package at http://bioconductor.org/packages/rrvgo/ and released under the GPL-3 License. The version of the software used in this article (rrvgo 1.10.0, Bioconductor 3.16) is also available in the Extended Data Section.

## Extended Data

Description: Source Package. Resource Type: Software. DOI: 10.22002/xa9g7-5mm38
